# Optimized Recovery of Immature Germ Cells after Prepubertal Testicular Tissue Digestion and Multi-Step Differential Plating: A Step towards Fertility Restoration with Cancer-Cell-Contaminated Tissue

**DOI:** 10.3390/ijms25010521

**Published:** 2023-12-30

**Authors:** Sven De Windt, Dhoha Kourta, Marc Kanbar, Christine Wyns

**Affiliations:** 1 Laboratoire d’andrologie, Pôle de Recherche en Physiologie de la Reproduction, Institut de Recherche Expérimentale et Clinique (IREC), Université Catholique de Louvain, 1200 Brussels, Belgium; sven.dewindt@uclouvain.be (S.D.W.); dhoha.kourta@uclouvain.be (D.K.); marc.kanbar@uclouvain.be (M.K.); 2 Department of Gynecology-Andrology, Cliniques Universitaires Saint-Luc, 1200 Brussels, Belgium

**Keywords:** immature testicular tissue, childhood cancer, testicular cells, enzymatic digestion, spermatogonial stem cells, spermatogonia, fertility preservation, differential plating, Poly-D-Lysine, ECM-based coating

## Abstract

Undifferentiated germ cells, including the spermatogonial stem cell subpopulation required for fertility restoration using human immature testicular tissue (ITT), are difficult to recover as they do not easily adhere to plastics. Due to the scarcity of human ITT for research, we used neonatal porcine ITT. Strategies for maximizing germ cell recovery, including a comparison of two enzymatic digestion protocols (P1 and P2) of ITT fragment sizes (4 mm^3^ and 8 mm^3)^ and multi-step differential plating were explored. Cellular viability and yield, as well as numbers and proportions of DDX4+ germ cells, were assessed before incubating the cell suspensions overnight on uncoated plastics. Adherent cells were processed for immunocytochemistry (ICC) and floating cells were further incubated for three days on Poly-D-Lysine-coated plastics. Germ cell yield and cell types using ICC for SOX9, DDX4, ACTA2 and CYP19A1 were assessed at each step of the multi-step differential plating. Directly after digestion, cell suspensions contained >92% viable cells and 4.51% DDX4+ germ cells. Pooled results for fragment sizes revealed that the majority of DDX4+ cells adhere to uncoated plastics (P1; 82.36% vs. P2; 58.24%). Further incubation on Poly-D-Lysine-coated plastics increased germ cell recovery (4.80 ± 11.32 vs. 1.90 ± 2.07 DDX4+ germ cells/mm^2^, respectively for P1 and P2). The total proportion of DDX4+ germ cells after the complete multi-step differential plating was 3.12%. These results highlight a reduced proportion and number of germ cells lost when compared to data reported with other methods, suggesting that multi-step differential plating should be considered for optimization of immature germ cell recovery. While Poly-D-Lysine-coating increased the proportions of recovered germ cells by 16.18% (P1) and 28.98% (P2), future studies should now focus on less cell stress-inducing enzymatic digestion protocols to maximize the chances of fertility restoration with low amounts of cryo-banked human ITT.

## 1. Introduction

Unlike young adolescents and men who can benefit from semen cryopreservation for fertility preservation before gonadotoxic therapies to cure cancer, for prepubertal boys who do not have complete germ cell maturation yet, cryopreservation of immature testicular tissue (ITT) containing undifferentiated germ cells including spermatogonial stem cells is the only option [1,2,3,4,5,6]. Follow-up studies already emphasized cryopreservation of human ITT to be well accepted, feasible and safe in the short term [7] and long term [8,9,10].

It was shown that 37% of testicular biopsies from prepubertal leukemia or lymphoma patients were contaminated by cancer cells [11] and transplantation of as few as 20 leukemic cells introduced cancer relapse in rats [12]. Approaches to restore fertility with cryo-banked ITT that circumvent the risk of reintroducing cancer cells back to the patients are urgently awaited as patients who participated in pilot fertility preservation programs during childhood have now reached the reproductive age and in vitro maturation with generation of functional sperm has so far not been achieved with human ITT [4]. However, transplanting isolated testicular cells, provided that the cell suspension is free of cancer cells, or creating a testicular organoid [13,14] using sorted single cells free of malignant cells followed by autografting could be considered.

One of the greatest and most consistent difficulties is to achieve a high germ cell recovery. Indeed, undifferentiated germ cells are present in very low proportions. These range from 1% germ cells in human ITT from newborns (2 and 7 days old) [15] to 3–4% of state 0–1 undifferentiated spermatogonia in 1-year and 7-year-old boys’ ITT [16]. Additionally, the spermatogonial quantity is disease and age-dependent, in particular, prepubertal hematological disorder patients (<7 years old) were identified to be at high risk for depleted spermatogonia following gonadotoxic therapy [17]. Methods to maximize germ cell recovery are therefore important.

To obtain cell suspensions from ITT with high purity and viability for all cell types, researchers from different teams worldwide have reported a plethora of methods, mainly with animal tissue. However, studies trying to maximize germ cell recovery from animal ITT [18] and studies reporting the impact of enzymatic digestion protocols on total or cell type-specific cellular yield and viability starting with human ITT are scarce [19,20]. In the study of Medrano et al. tissue digestion and cell isolation were performed but no germ cell quantification was reported [21] and in the study of Sadri-Ardekani et al. propagation of human spermatogonial stem cells was successfully performed in order to increase the colonization of the spermatogonial stem cell niche [22].

Furthermore, recovery of undifferentiated germ cells is also challenging because these cells are known to be highly vulnerable to exposure to cell stress-inducing conditions [23], including high concentrations of digestion enzymes [24], and do not properly adhere to uncoated plastic dishes [25].

So far, the majority of studies aimed at germ cell recovery or enrichment rely on the adherence property of testicular somatic cells to uncoated plastic dishes for their elimination and do not consider the fraction of germ cells that adhere to such dishes and are thus discarded [25,26]. Germ cell-enriched populations thus only originate from the second plating as reported in experiments using human adult testicular tissue [27,28].

Goel et al. identified Poly-D-Lysine as a facilitator for the adherence of floating germ cells that could not be adhered to uncoated plastics [26]. However, when used in a two-step plating strategy, only a 20% enrichment was achieved and quantification was not evaluated [18].

Therefore, the objectives of this study were first to compare two enzymatic digestion protocols based on the hypothesis that long incubation times and high concentrations of collagenase IV could have deleterious effects on the recovery of germ cells [24], and second, to determine the proportion of germ cells recovered at the different steps of a multi-step plating strategy and to identify a technique that allows the maximal recovery of germ cells present in neonatal testes at day 3, i.e., mainly immature germ cells including the spermatogonial stem cell population.

Due to the scarcity of human prepubertal ITT available for research, we used neonatal porcine testicular tissue. To better support the appropriateness of this surrogate model, we further characterized the neonatal porcine tissue in a first experiment and compared it to available data for human ITT.

## 2. Results

### 2.1. Weight/Volume Ratio and Tissue Histology of Neonatal Porcine ITT

The average weight of the 12 testes was 611.37 ± 121.74 mg. The average volume of the 12 testes was 629.17 ± 169.84 µL. The weight/volume ratio obtained was 1.00 ± 0.15 g/cm^3^ (Table S1). Based on Hematoxylin and Eosin (H&E) staining, we identified all testes to have a normal architecture with germ cells and Sertoli cells inside seminiferous tubules surrounded by interstitial Leydig cells and peritubular myoid cells.

### 2.2. Effects of Testicular Fragment Size and Enzymatic Digestion Protocol on Total Cellular Viability, Concentration and Yield Directly after Digestion

Total cell viability, concentration and yield directly after digestion are presented in Table 1. Total cell viability was in all conditions > 92%. The highest cellular yields normalized per mg of digested ITT were obtained using P1 for 8 mm^3^ fragments, and using P2 for 4 mm^3^ fragments, though there were no statistically significant differences.

### 2.3. Effect of Testicular Fragments Size and Enzymatic Digestion Protocol on DDX4+ Germ Cell Numbers and Proportions Directly after Digestion

Pooled results for both fragment sizes and digestion protocols revealed the proportion of DDX4+ germ cells, directly after digestion, to be 4.51%. Proportion analysis directly after digestion using P1 revealed 6.35 ± 1.39% of all testicular cells to be DDX4+ germ cells. In contrast, DDX4+ germ cells account for 2.67 ± 0.09% of all testicular cells using P2 (*p* = 0.11).

Quantification of DDX4+ germ cells normalized per mg of digested ITT directly after digestion revealed P2 as having a slightly higher number of recovered DDX4+ cells compared to P1 for the small fragments (19.66 ± 3.56 vs. 13.00 ± 1.33 DDX4+ germ cells/mg ITT, respectively). In addition, results for P1 revealed a higher recovery of germ cells normalized per mg of digested ITT for the bigger fragments compared to P2 (43.33 ± 14.44 vs. 9.67 ± 2.44 DDX4+ germ cells/mg ITT, respectively).

### 2.4. Effects of Testicular Fragment Size and Enzymatic Digestion Protocol on Cell Type-Specific Cellular Numbers and Yield after the Complete Differential Plating Procedure

**Figure 1:**
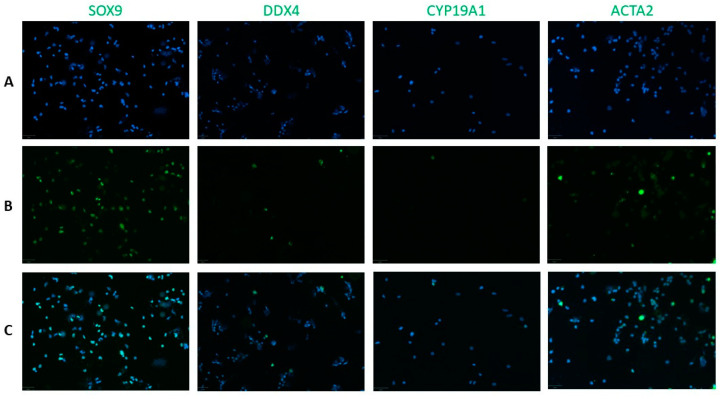
Representative images of SOX9, DDX4, CYP19A1 and ACTA2 immunofluorescence analysis of adherent cell fractions on uncoated plastics. (**A**) Hoechst33342. (**B**) Cell type-specific marker. (**C**) Merged image. Scale-bars = 50 µm.

Adherent cells 24 h after incubation on uncoated plastics (Figure 1), floating cells that were subsequently incubated three days on Poly-D-Lysine-coated plastics and remaining floating cells were analyzed using immunocytochemistry (ICC) to investigate the presence and numbers of all testicular cell types.

**Figure 2:**
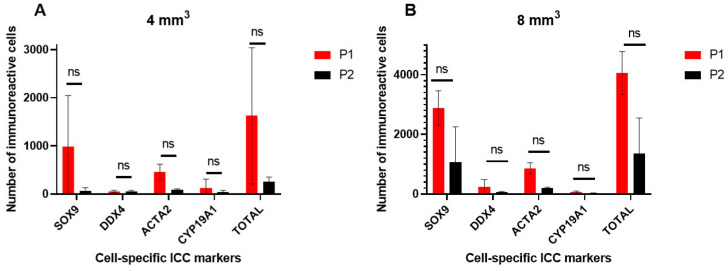
Comparison of both enzymatic digestion protocols for equal-size testicular fragments after the complete multi-step differential plating strategy. (**A**) Mean number of immunoreactive cells for each cell type-specific marker (SOX9: Sertoli cells; DDX4: germ cells; ACTA2: peritubular myoid cells; CYP19A1: Leydig cells; TOTAL: total number of immunoreactive cells for the four tested markers) per donor using 4 mm^3^ neonatal porcine testicular tissue fragments. (**B**) Mean number of immunoreactive cells for each cell type-specific marker per donor using 8 mm^3^ neonatal porcine testicular tissue fragments. ns: non-significant, *n* = 3.

When comparing digestion protocols for equal fragment size, after the complete multi-step differential plating strategy, results revealed no statistically significant differences in cell numbers for any of the cell types (Figure 2A,B).

**Figure 3:**
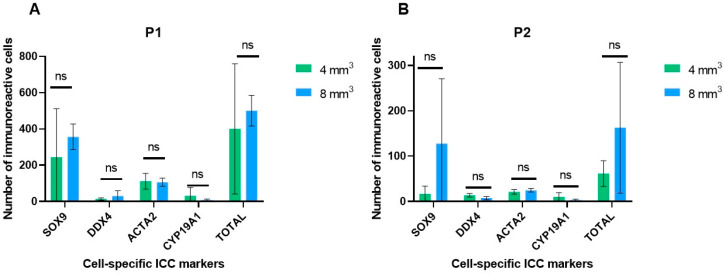
Comparison of the two different testicular fragment sizes on numbers of immunoreactive cells normalized per mg and per protocol after the complete multi-step differential plating strategy. (**A**) Mean number of immunoreactive cells for each cell type-specific marker (SOX9: Sertoli cells; DDX4: germ cells; ACTA2: peritubular myoid cells; CYP19A1: Leydig cells; TOTAL: total number of immunoreactive cells for the four tested markers) using enzymatic digestion protocol P1. (**B**) Mean number of immunoreactive cells for each cell type-specific marker and total immunoreactive cells using enzymatic digestion protocol P2. ns: non-significant, *n* = 3.

When comparing both fragment sizes after the complete differential plating strategy, results normalized per mg of digested tissue for each digestion protocol revealed no statistically significant differences in cell numbers for any of the cell types (Figure 3A,B).

Altogether, grouped results for both fragment sizes revealed the total number of immunoreactive cells for the four markers tested at the end of the multi-step differential plating strategy, normalized per mg of digested tissue, to be three-fold higher using P1 compared to P2 (5684.67 ± 1413.17 vs. 1606.00 ± 1299.67 immunoreactive cells/donor, respectively). Pooled results for both fragment sizes and digestion protocols revealed 3.12% of all testicular cells at the end of the multi-step differential plating strategy to be DDX4+ germ cells.

ICC for DDX4+ cells revealed no statistically significant differences when comparing either the digestion protocols or the fragment sizes. For 4 mm^3^ ITT fragments, P2 revealed a slightly higher number of DDX4+ germ cells recovered compared to P1 (57.67 ± 18.56 vs. 53.00 ± 25.94 DDX4+ germ cells/fragment, respectively) (Figure 2A). For 8 mm^3^ ITT fragments, P1 revealed a higher recovery number of DDX4+ germ cells compared to P2 (239.33 ± 236.90 vs. 59.67 ± 28.87 DDX4+ germ cells/fragment, respectively) (Figure 2B). Quantification of DDX4+ germ cells normalized per mg of digested ITT confirmed the same trends; P2 had a slightly higher number of recovered DDX4+ cells compared to P1 for the small fragments (13.63 ± 3.90 vs. 12.94 ± 6.72 DDX4+ germ cells/mg ITT, respectively) (Figure 3A,B) and P1 revealed a higher recovery of germ cells normalized per mg of digested ITT for the bigger fragments compared to P2 (29.38 ± 28.75 vs. 7.24 ± 3.44 DDX4+ germ cells/mg ITT, respectively) (Figure 3A,B).

Quantification of the other testicular cell types per digestion protocol for the two fragment sizes is shown in Figure 3A,B. Although not statistically different, a trend for higher numbers of SOX9+ and ACTA2+ cells normalized per mg was found with P1 for both fragment sizes.

### 2.5. Evaluation of the Multi-Step Differential Plating Procedure to Increase DDX4-Positive Germ Cell Recovery

**Figure 4:**
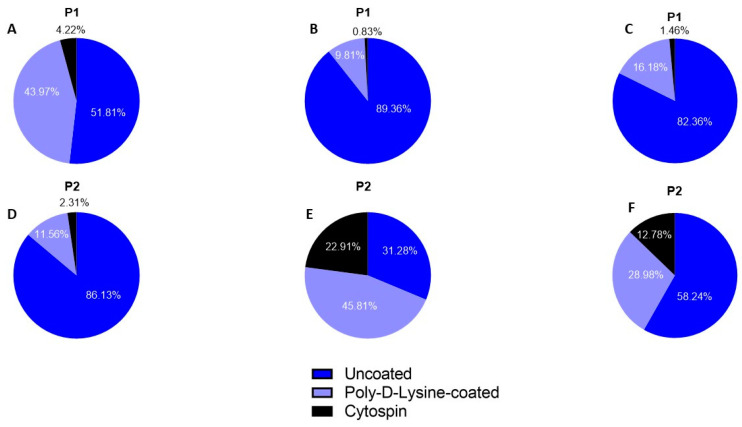
Proportion of recovered DDX4+ germ cells at each step of the multi-step differential plating strategy. (**A**–**C**) Proportions obtained using enzymatic digestion protocol P1 for 4 mm^3^, 8 mm^3^ and pooled fragment sizes respectively, on a pooled total of 890 DDX4+ germ cells. (**D**–**F**) Proportions obtained using enzymatic digestion protocol P2 for 4 mm^3^, 8 mm^3^ and pooled fragment sizes respectively, on a pooled total of 352 DDX4+ germ cells.

Proportion analysis of recovered DDX4+ cells after enzymatic digestion with P1 and differential plating for 4 mm^3^ fragments revealed 51.81% (86/166) of DDX4+ cells to adhere to uncoated plastics. In addition, 43.97% (73/166) of DDX4+ cells were recovered after the three days of Poly-D-Lysine-coated plastics incubation (Figure 4A). For 8 mm^3^ fragments, corresponding figures were 89.36% (647/724) and 9.81% (71/724) (Figure 4B).

Proportion analysis of recovered DDX4+ cells after enzymatic digestion with P2 and differential plating for 4 mm^3^ fragments revealed that 86.13% (149/173) of DDX4+ cells adhered to the uncoated plastic dishes, whereas an additional 11.56% (20/173) were recovered using Poly-D-Lysine-coated plastics (Figure 4D). For 8 mm^3^ fragments, 31.28% (56/179) DDX4+ cells adhered to uncoated dishes and 45.81% (82/179) adhered to Poly-D-Lysine-coated dishes (Figure 4E).

At the end of the multi-step differential plating procedure, 4.22% (7/166) and 0.83% (6/724) of all DDX4+ germ cells remained floating when P1 was used for digestion of 4 mm^3^ and 8 mm^3^ fragments, respectively (Figure 4A,B). When P2 was used for the digestion of 4 mm^3^ and 8 mm^3^ fragments, 2.31% (4/173) and 22.91% (41/179) of all DDX4+ germ cells remained floating, respectively (Figure 4D,E).

Figure 4 shows DDX4+ germ cell proportions obtained with digestion protocol P1 (Figure 4A–C) and P2 (Figure 4D–F) at the different steps of the procedure. No statistically significant differences could be identified.

Table 2 shows the proportions of DDX4+ germ cells among the total testicular cell population that adhered at each step of the multi-step differential plating. The results revealed enrichment of DDX4+ germ cells in the Poly-D-Lysine-coated plastic-adherent germ cell fraction for both 4 mm^3^ and 8 mm^3^ ITT fragments, independent of enzymatic digestion protocol, except for the 4 mm^3^ ITT fragments digested with P2, where only 5.55 ± 4.41% of all Hoechst33342+ adherent fractions were DDX4+ germ cells.

### 2.6. Evaluation of Enzymatic Digestion Protocols in Regards to DDX4+ Germ Cell Numbers along and after the Complete Differential Plating Procedure

The sum of all obtained DDX4+ germ cells for both tissue fragment sizes revealed an average 4.80 ± 11.32 DDX4+ germ cells/mm^2^ to be recovered using P1 compared to 1.90 ± 2.07 DDX4+ germ cells/mm^2^ using P2. A detailed analysis of these obtained DDX4+ cells revealed an average 11.86 ± 18.38 DDX4+ germ cells/mm^2^ to be recovered by adhesion to uncoated plastic dishes using P1 compared to an average 3.32 ± 2.49 DDX4+ germ cells/mm^2^ using P2. Poly-D-lysine-coated plastic dishes were able to obtain an additional adherence of 2.33 ± 2.33 and 1.65 ± 1.82 DDX4+ germ cells/mm^2^ using P1 and P2, respectively. At the end, 0.21 ± 0.29 DDX4+ germ cells/mm^2^ remained floating using P1 compared to 0.73 ± 0.97 DDX4+ germ cells/mm^2^ using P2. No statistically significant differences were found when comparing procedures with P1 and P2.

## 3. Discussion

Studies aimed at germ cell recovery have combined different cell-enrichment strategies such as silica-based [26,29] or non-particulate media [30,31] density gradients and differential plating methods. Although germ cell-enriched suspensions with purities > 80% were obtained, quantification of immature germ cell loss due to the technique has been investigated only for non-particulate media gradients, revealing a loss of 46.72 ± 4.69% of all germ cells contained in the tissue [31]. This points to the need to find methods able to maximize germ cell yields. In differential plating strategies, one of the ECM-based coatings identified to facilitate the adherence of immature porcine germ cells is Poly-D-Lysine [26]. Poly-D-Lysine or Poly-L-Lysine, a small natural homopolymer of the essential amino acid (D-Lysine or L-Lysine, respectively) of ECM proteins [32] is either applied in a first step [26,30,31] or in a second step after gelatin-coated plastic incubation to remove somatic cells [29]. We used Poly-D-Lysine, rather than Poly-L-Lysine as it was shown to be resistant to protease breakdown [33], to explore its applicability in maximizing germ cell recovery. This is of specific importance given the fact that in both digestion protocols, trypsin, a known serine protease, is used. Of note also, murine germ cells appeared to attach to uncoated plastics [34], raising the possibility to optimize germ cell recovery using the attached fraction. Ideally, germ cells that are discarded, i.e., those contained in the initial uncoated plastic-adherent cell fraction and in the remaining floating fraction after differential plating, should be considered in the recovery procedure in the context of progressing towards fertility restoration strategies with cancer cell-contaminated ITT.

While the development of fertility preservation measures for prepubertal boys is an urgent matter, ITT of these boys is hardly available for research purposes. We therefore used neonatal porcine tissue. Initial characterization of this tissue showed many similarities with human ITT, especially regarding the weight/volume ratio and tissue architecture. We obtained a weight/volume ratio of 1.00 ± 0.15 g/cm^3^ using 12 neonatal porcine testes, which is highly comparable to 1.0 g/cm^3^ reported when analyzing autopsy-derived testes from 10 young boys [35]. The 4.51% germ cell proportion observed after digestion of porcine ITT is also similar to proportions observed in human samples. Indeed, single-cell transciptomic studies using human neonatal and prepubertal tissue indicate that spermatogonia and particularly the undifferentiated are present in very low proportions ranging from 1% germ cells [15] to 3–4% state 0–1 undifferentiated spermatogonia in 1-year and 7-year-old boys’ ITT [16]. Such low proportions call for finding ways to maximise undifferentiated germ cell recovery for downstream research in the field of fertility preservation and restoration in prepubertal boys.

Our findings, together with other advantages of using porcine material for research on fertility restoration methods, e.g., easy access to castration-derived testes in large quantities providing a reliable and constant source of testicular material [36], piglets having an extended prepubertal period by contrast with rodent models [37] and data on reproductive processes in pig [38,39,40] highlight that piglet testicular tissue can be considered an appropriate alternative experimental material that is highly representative of human prepubertal testicular tissue.

In vivo approaches to restore fertility using limited available amounts of thawed cancer cell-contaminated tissue imply that testicular cells are preselected before being used for autotransplantation of either a cancer cell-cleared germ cell suspension or as a cancer cell-free testicular organoid containing all testicular cell types. To that end, gathering the highest number of viable germ cells is crucial as, currently, propagation of spermatogonia seems difficult due to significant germ cell loss over culture time [21,41,42,43,44,45,46], except in one study where propagation of human spermatogonial stem cells was successfully performed in order to increase the colonization of the spermatogonial stem cell niche [22]. However, for clinical application it is preferable to avoid cell modification that can occur after prolonged culture. We found the proportion of germ cells to be 4.51% directly after digestion and 3.12% after the whole multi-step plating procedure, corresponding to a reduced proportion of germ cells lost with our differential plating strategy when compared to previously reported germ cell yields [31].

Focussing on tissue digestion protocols could further improve germ cell yields. The current literature does not allow for comparing the efficacy of enzymatic digestion protocols, not directly after digestion nor after germ-cell enrichment strategies, as often only proportions of recovered cells are reported rather than cell numbers per weight unit of digested tissue. We compared a commonly used enzymatic digestion protocol (P1) reported in the literature [47] with an in-house used protocol (P2). P2 was modified from Dores et al. 2015 [48], by increasing the concentration of hyaluronidase to allow for more efficient digestion of the testicular connective tissue, taking into account the different composition of prepubertal tissue compared to adult tissue [49,50,51] and by removing the addition of DNase at the end of the digestion procedure as no cell clumping due to free DNA was seen in earlier preliminary experiments. Directly after digestion, we did not find any superiority of one digestion protocol over the other in terms of total numbers of viable cells and cell yields.

The average number of cells recovered per mg of digested tissue with P1 and P2 directly after digestion was also higher than previously reported. Indeed, a study reporting total cell numbers directly after enzymatic digestion of prepubertal human ITT obtained 12,381 testicular cells per mg of digested tissue [19], whereas we obtained between 340,000–380,000 ± 90,000 testicular cells per mg of digested tissue using P1 for 4 mm^3^ and 8 mm^3^, respectively. When P2 was used for both fragment sizes, we obtained 460,000 ± 100,000 testicular cells per mg of digested tissue for 4 mm^3^ fragments and 350,000 ± 90,000 testicular cells per mg of digested tissue for 8 mm^3^ fragments (Table 1). Lower yields of testicular cells [19] can be explained by a one-minute sedimentation applied after the first enzymatic digestion step followed by removal of the supernatant, potentially still containing high concentrations of testicular cells, while in our study centrifugation to concentrate cells into pellets before supernatant removal was performed in both protocols.

Starting the digestion with an optimal fragment size with regards to digestion outcomes also matters as it may influence the penetration efficacy of the enzymes. Although comparisons between fragments 4 and 8 mm^3^ in size were not statistically significant, we observed a trend of P1 having a higher efficacy for bigger (8 mm^3^) fragments and P2 having a higher efficacy for smaller (4 mm^3^) fragments, suggesting that some digestion protocols may be more suitable for smaller fragment sizes, as those are most often available in clinical fertility preservation programs.

In our study, we showed that the majority of DDX4+ cells were capable of adhering to uncoated plastic dishes and that 16–29% of all DDX4+ cells remained floating after overnight incubation and could further adhere to the dish by using Poly-D-Lysine-coated plastics. These results highlight the importance of recovering germ cells at each step during a multi-step differential plating procedure. Recovering adherent germ cells from uncoated plastics is a strategy to maximize germ cell recovery. Considering that testicular cell culture requires frequent culture medium changes, our study gave insight into the proportion of precious floating DDX4+ germ cells that, without differential plating, would potentially have been lost. In total, 82.36% (P1) and 58.24% (P2) of all DDX4+ germ cells were found to adhere to the uncoated dish in the first plating, which was to the best of our knowledge not evaluated in previous studies and points to the need to develop methods to recover this precious fraction. Either mild Accutase, as used by Dong et al. to harvest spermatogonial stem cell-like clusters [52], or trypsin [53] could be considered. However, Accutase seems a better candidate enzyme, as a comparison using murine testicular cells revealed higher total numbers of CD90+ spermatogonial stem cells to be obtained compared to trypsin [54].

In addition to the germ cell population, we also explored if enzymatic digestion protocols could potentially have a negative impact on other testicular cell types. Although not statistically significant different, we observed that some cell types (e.g., SOX9, ACTA2) seemed to be more sensitive to enzymatic digestion using P2. In addition to multi-step exposure to enzymes and higher enzyme concentrations with longer exposure times, P2 also included two wash steps requiring centrifugation that potentially increased cell stress. Such conditions could be more prone for damaging testicular cells, notably by alterations of membrane receptors [55,56]. Our results showed that P1 allowed the recovery of a three-fold higher total number of immunoreactive cells for all four markers tested normalized per mg of digested tissue, identifying P1 to be less harmful for the cells, though the mechanism involved, e.g., direct membrane receptor damage, was not investigated in this study. Future experiments should focus on investigating if higher enzyme concentrations and incubation times can cause membrane receptor damage, as these receptors will be important for downstream applications such as in vitro spermatogenesis. Of note, directly after digestion, no significant differences could be identified regarding cellular viability nor yield, potentially as no immediate cellular death but rather cell damage was created. These damaged cells then probably died during the differential culture steps, explaining the three-fold difference in total immunoreactive cell numbers at the end of the multi-step differential plating procedure.

Knowing the total germ cell numbers recovered per mg of digested tissue directly after digestion and at the end of the whole procedure is crucial to allow continuation of research, as this allows researchers to select the best-adapted protocol in terms of cell yield and cell stress for downstream applications. Using the obtained testicular cell fractions, future studies should focus on understanding why some undifferentiated germ cells adhere to uncoated and coated plastic dishes. To this end, characterization of germ cell subpopulations using transcriptomics as recently reported by Guo et al. [57] should be used to identify in which fraction the spermatogonial stem cells are present.

Although this preliminary study adds knowledge to the existing literature regarding germ cell recovery from ITT, some limitations have to be considered while interpreting the results. Despite the fact that the literature has demonstrated that no significant differences have been obtained in terms of total cellular yield nor viability when using tissue derived from non-littermates [58], some variability in tissue content cannot be excluded and our data obtained with three tissue donors should be further confirmed. While assessment of differences in the suspension cell population between the first and second differential plating steps was not performed in this set of experiments, further studies should explore this aspect.

## 4. Materials and Methods

### 4.1. Experimental Design

**Figure 5:**
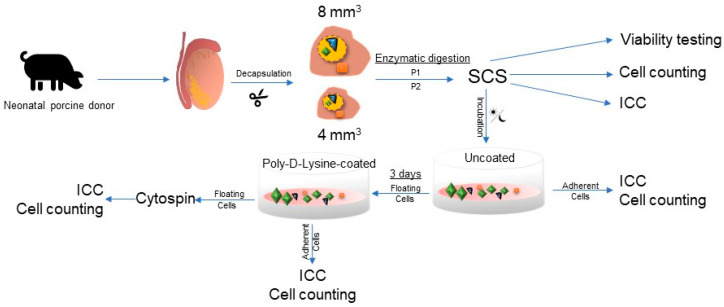
Graphical representation of the study design. SCS: single-cell suspension; ICC: immunocytochemistry.

This study was conducted as graphically represented in Figure 5. Briefly, on day 1, testes from three animals were minced into fragments of 4 mm^3^ and 8 mm^3^ (sizes representative of human prepubertal cryopreserved biopsies for fertility preservation) [59,60], prior to being digested with either a commonly used digestion protocol (P1) [47] or with an in-house used protocol slightly modified from Dores et al. [48] (P2). After digestion, the testicular cell suspensions obtained for these four conditions were analyzed to determine the total cellular yield, viability, absolute numbers and proportions of DDX4+ germ cells. Two hundred µL of each cell suspension (containing one million viable cells/mL) were incubated overnight on uncoated plastic dishes. On day 2, adherent cells were recovered for ICC analysis and floating cells were incubated for three days on Poly-D-Lysine-coated plastic dishes. At the end of the Poly-D-Lysine-coated plastic incubation on day 5, adherent cells were recovered for ICC analysis and remaining floating cells were attached to glass slides for ICC analysis using centrifugation cytospin. The target population, immature germ cells including the spermatogonial stem cell population, were identified by DDX4. All experiments were conducted in triplicate.

### 4.2. Animals

Testes from piglets aged 3 and 5 days were recovered from a local Belgian swine farm. As neonatal porcine testes are a byproduct of castration, the Belgian law approves the research on porcine testes without prior ethical assessment. Testes were transported to the lab at 4 °C in DMEM/F12 (ThermoFisher Scientific, Ghent, Belgium, 11330032) containing 10 units/mL of penicillin and 10 µg/mL of streptomycin (ThermoFisher Scientific, Ghent, Belgium, 15140122). Fresh piglet testes with normal age-matched physiological volumes were used for the characterization of the porcine tissue (*n* = 12 donors) and for tissue digestion experiments (*n* = 3 donors). Upon arrival at the lab, testes were decapsulated to remove the epididymis prior to weighing, volume determination and further analyses.

### 4.3. Characterization of Neonatal Porcine Testicular Tissue

Twelve testes derived from 4 litters, ages ranging from 3 (*n* = 9) to 5 days (*n* = 3), were used to determine the tissue weight-to-volume ratio and proportions of germ cells. Testes’ volumes were determined using the water displacement method and their weight was measured with an ultra-precision balance (A&D Company Limited, Tokyo, Japan, HR-150AZ) before removing the tunica albuginea. After tissue digestion using both P1 and P2, germ cell proportions were calculated by dividing the number of recovered DDX4+ cells by the total number of Hoechst33342+ cells.

### 4.4. Tissue Handling before Tissue Digestion and Enzymatic Digestion Protocols

Testicular tissue was minced into fragments of 4 mm^3^ or 8 mm^3^. In addition, a small testicular fragment of each testis was fixed overnight in 4% formaldehyde (VWR Chemicals, Leuven, Belgium, 17A184141) followed by paraffin embedding for H&E staining and ascertaining the normality of the tissue histology and more specifically the presence of germ cells. To allow normalization of outcome parameters for fragment size, the weight of each digested fragment was determined by using an Ultra-Precision Balance (A&D Company Limited, Tokyo, Japan, HR-150AZ). Enzymatic digestion was performed for P1 as described elsewhere [47]. Briefly, for P1, testicular tissue fragments were digested with 1 mg/mL Collagenase IV (Sigma-Aldrich, Overijse, Belgium, C5138) for 10 min at 37 °C, followed by addition of 0.25% Trypsin/EDTA (ThermoFisher Scientific, Ghent, Belgium, 252000056) and 1.4 mg/mL DNase (Merck, Overijse, Belgium, 11284932001) for 10 min at 37 °C. For P2, fragments were digested with 2 mg/mL Collagenase IV (Sigma-Aldrich, Overijse, Belgium, C5138) for 40 min at 37 °C, followed by addition of 1 mg/mL Hyaluronidase (Sigma-Aldrich, Overijse, Belgium, H1115000) for 20 min at 37 °C, prior to addition of 0.25% Trypsin/EDTA (ThermoFisher Scientific, Ghent, Belgium, 252000056) for 10 min at 37 °C. Enzymatic reaction was stopped, in both protocols, by addition of 10% fetal bovine serum (Sigma-Aldrich, Overijse, Belgium, F7524) and filtered through a 70 µM cell-strainer (PluriSelect Life Science, Leipzig, Germany, 43-50070-01). Cells were resuspended in 2 mL of culture medium. A small fraction of the cell suspensions was used for immediate total cellular yield and viability measurements, another fraction was used for differential plating experiments.

### 4.5. Cellular Numbers, Yield and Viability

Total cellular yield and viability were determined by trypan blue exclusion testing directly after digestion for each condition. Briefly, 10 µL of fresh digested cell suspension was mixed 1:1 with trypan blue (Sigma-Aldrich, Overijse, Belgium, T8454) and added to a 0.0025 mm^2^ Bürker hemocytometer chamber. Comparison of cellular yield and viability for both fragment sizes was performed by normalizing the cellular counts per mg of digested testicular tissue. Only viable cells were considered when reporting total cell concentrations and yields in cell suspensions. Cell type-specific yields were defined as the number of recovered immunoreactive cells for each cell-specific marker during culture and normalized per mg of digested tissue.

### 4.6. Preparation of Poly-D-Lysine-Coated Plastic Dishes, Cell Culture and Differential Plating Procedure

Poly-D-Lysine-coated plastic dishes were obtained by incubation of uncoated plastic dishes with 50 µg/mL Poly-D-Lysine for 1 h followed by three rinse steps using distilled water and drying for 3 h, according to the manufacturer’s recommendations (ThermoFisher Scientific, Ghent, Belgium, A3890401).

Freshly digested cells were seeded at 200,000 cells/well on uncoated chambered wells (Proxylab, Beloeil, Belgium, 80841) in 200 µL of DMEM/F12 (ThermoFischer Scientific, Ghent, Belgium, 11330032) containing 10% fetal bovine serum (Sigma-Aldrich, Overijse, Belgium, F7524), 10 units/mL of penicillin and 10 µg/mL of streptomycin (ThermoFisher Scientific, Ghent, Belgium, 15140122). Single-cell suspensions were incubated overnight at 35 °C in 5% CO_2_. On day 2, adherent cells were processed for ICC while floating cells were incubated on Poly-D-lysine-coated chambered wells (Proxylab, Beloeil, Belgium, 80841) for 3 days at 35 °C in 5% CO_2_. On day 5, adherent cells were processed for ICC and remaining floating cell fractions were attached onto glass slides (Superfrost^TM^ Plus Adhesion Microscope Slides, Epredia, Breda, the Netherlands, J1800AMNZ) using cytospin centrifugation (Shandon Scientific Limited, Runcorn, United Kingdom, 74000101) for ICC processing.

### 4.7. Immunocytochemistry and Immunostained Cells Quantifications

Immunocytochemistry was performed to evaluate the presence and proportion of DDX4+ germ cells directly after digestion and the presence and proportion of all testicular cell types in each step of the multi-step differential plating procedure. For germ cells, the numbers of DDX4+ cells and proportions calculated as the ratio between the DDX4+ germ cell numbers and the total number of Hoechst33342-labelled cells directly after digestion were recorded. During the differential plating strategy, DDX4+ germ cell numbers obtained at each step of the differential plating and the total number of DDX4+ cells recovered from the complete differential plating procedure were recorded. Directly after digestion, single-cell suspensions were fixed in 4% paraformaldehyde (VWR Chemicals, Leuven, Belgium, 17A184141) for 15 min, mixed with 2% agar prior to being embedded in paraffin and 5 µm sections preparation. Sections were deparaffinized and rehydrated in toluene and methanol baths. Endogenous peroxidase activity was blocked in 0.3% H_2_O_2_ for 30 min followed by antigen retrieval in citrate buffer for 50 min at 98 °C and washes in Tris-buffered saline (TBS)/Triton. Sections were incubated with TBS/Tween 0.05% (TBST) containing 5% bovine serum albumin (BSA) (Sigma-Aldrich, Overijse, Belgium, A7030) for 30 min at room-temperature in the dark to block nonspecific reactions followed by incubation of DDX4 (Table 3) diluted 1:500 in TBST/1% BSA for 90 min at room-temperature and washes in TBST. Secondary anti-rabbit antibody (Envision + system-labelled polymer-horseradish peroxidase; Agilent Technologies, California, CA, Santa Clara, United States, K4003) was added for 40 min at room-temperature and followed by washes in TBST. Antibody signal was detected using Alexa Fluor^TM^ 488 Tyramide (ThermoFisher Scientific, Ghent, Belgium, B40953) for 10 min at room-temperature and nuclei were counterstained using 10 µg Hoechst33342 (ThermoFisher Scientific, Ghent, Belgium, 62249) diluted in TBST/BSA 10%. After final washing in TBST and distilled water, slides were mounted using Dako fluorescence mounting medium and images were acquired by fluorescent microscopy (Zeiss, Zaventem, Belgium, LSM800) and quantification of nuclei and cells expressing DDX4 was performed on three randomly selected fields/section using QuPath 0.3.0 software [61]. During each step of the multi-step differential plating strategy, each cell type-specific marker was used in one chambered well per condition tested. Cells were fixed in 4% formaldehyde (VWR Chemicals, Leuven, Belgium, 17A184141) for 5 min prior to permeabilization using 0.1% triton in phosphate-buffered saline (PBS) (Lonza, Verviers, Belgium, BE17-516F) for 10 min. Cells were incubated for 1 h in PBS-triton (PBST) containing 5% (BSA) (Sigma-Aldrich, Overijse, Belgium, A7030) to block non-specific bindings. Primary antibodies (Table 3), diluted in PBST containing 1% BSA, were added to the cells and incubated overnight at 4 °C. The next day, secondary anti-rabbit or anti-mouse antibodies (Envision + system-labeled polymer-horseradish peroxidase; Agilent Technologies, California, CA, Santa Clara, United States, K4003 or K4001) were incubated for 1 h at room-temperature. Following washing in PBST, nuclei were counterstained using 200 µL of Hoechst33342 (ThermoFisher Scientific, Ghent, Belgium, 62249) diluted (1:1000) in PBST containing 10% BSA. After final washes in PBS, slides were mounted using Dako fluorescent mounting medium (Agilent Technologies, California, CA, Santa Clara, United States, S3023). A negative control, omitting the primary antibody, was used. Images were acquired by fluorescent microscopy (Zeiss, Zaventem, Belgium, LSM800) and quantification of nuclei and cells expressing SOX9, DDX4, ACTA2 and CYP19A1 was performed on three randomly selected fields/section using QuPath 0.3.0 software [61]. Cell numbers were normalized per mg of digested tissue to allow comparisons.

### 4.8. Statistical Analysis

Data presentation and statistical analysis were performed using GraphPad Prism, version 5.0 (GraphPad Software, La Jolla, CA, USA). Data are presented as mean ± SD. Normality and homogeneity of variance were assessed using Levene’s test. Comparisons between fragment sizes and enzymatic digestion protocols were evaluated using the parametric Student’s *t*-test. In case the data showed a non-normal distribution, the non-parametric Mann–Whitney test was used. Significant differences were statistically set at α = 0.05 and represented as * (*p* ≤ 0.05) on the graphs.

## 5. Conclusions

Altogether, this study added data to support the appropriateness of using neonatal porcine testicular tissue as a surrogate model for scarce human ITT. To the best of our knowledge, we are the first to report the weight/volume ratio of neonatal porcine testes and point to a similar proportion of germ cells in human prepubertal ITT. Moreover, for the first time, immature germ cells were found to attach to uncoated plastics. Our data further highlighted the benefit of a multi-step differential plating strategy to obtain a higher recovery of immature germ cells as no considerable germ cell loss due to the multi-step plating strategy was identified. As spermatogonia are very scarce, this study offered insight into methods such as differential plating using Poly-D-Lysine-coated plastics and showed it to be of benefit for increasing the recovery of germ cells. Future studies should focus on identifying optimal proteins that can be used for plastic coating to further increase germ cell recovery. For prepubertal cancer survivors facing impaired fertility at reproductive age, the number of recovered germ cells from banked tissue is of utmost importance as, so far, all outcome data from fertility restoration options with human prepubertal ITT showed germ cell loss to be the main limiting factor.

## Figures and Tables

**Table 1:** Measurements of cellular viability, concentration and yield for both fragment sizes and enzymatic digestion protocols directly after digestion.

Fragment Volume	Viability (%)	Concentration (Cells × 10^6^/mL)	Yield (Cells × 10^6^/mg)
Protocol P1			
4 mm^3^	92.06 ± 2.22	0.71 ± 0.20	0.34 ± 0.09
8 mm^3^	95.06 ± 0.54	1.52 ± 0.33	0.38 ± 0.09
Protocol P2			
4 mm^3^	94.59 ± 1.91	0.95 ± 0.14	0.46 ± 0.10
8 mm^3^	94.22 ± 0.41	1.46 ± 0.40	0.35 ± 0.09

**Table 2:** DDX4+ germ cell proportions among the total adherent Hoechst33342+ testicular cell population per enzymatic digestion protocol, fragment size and individual step of the multi-step differential plating strategy.

Fragment Size (mm^3^)	Individual Plating Step	DDX4+ Germ Cell Proportion among Total Adherent Cell Fraction (%)	Total Number of Adherent Hoechst33342+ Testicular Cells Recovered
Protocol 1			
4	Uncoated plastic-adherent cell fraction	1.85 ± 1.44	6365
	Poly-D-Lysine-coated plastic-adherent cell fraction	15.32 ± 19.44	418
	Cytospin-adherent cell fraction	3.86 ± 3.85	258
8	Uncoated plastic-adherent cell fraction	3.66 ± 2.77	13,829
	Poly-D-Lysine-coated plastic-adherent cell fraction	13.20 ± 10.14	537
	Cytospin-adherent cell fraction	1.59 ± 2.75	367
Protocol P2			
4	Uncoated plastic-adherent cell fraction	7.62 ± 7.71	3368
	Poly-D-Lysine-coated plastic-adherent cell fraction	5.55 ± 4.41	441
	Cytospin-adherent cell fraction	41.67 ± 38.19	11
8	Uncoated plastic-adherent cell fraction	0.35 ± 0.26	13,686
	Poly-D-Lysine-coated plastic-adherent cell fraction	16.96 ± 14.18	481
	Cytospin-adherent cell fraction	66.82 ± 33.11	97

**Table 3:** Antibodies used in the study.

Antibody	Reference	Manufacturer	Dilution	Target Cell
SOX9	Ab185966	Abcam, Cambridge, United Kingdom	1:2000	Sertoli cell
DDX4	Ab13840	Abcam, Cambridge, United Kingdom	1:2000	Germ cell
CYP19A1	Ab139492	Abcam, Cambridge, United Kingdom	1:2000	Leydig cell
ACTA2	A2547	Sigma-Aldrich, Overijse, Belgium	1:2000	Peritubular myoid cell

## Data Availability

Data are contained within the article and supplementary materials.

## Supplementary Materials

The supporting information can be downloaded at: https://www.mdpi.com/article/10.3390/ijms25010521/s1.

## Abbreviations

ACTA2	Actin alpha 2
BSA	Bovine serum albumin
CYP19A1	Cytochrome P450 Family 19 Subfamily A Member 1
DDX4	DEAD-Box Helicase 4
ECM	Extracellular matrix
FGFR3	Fibroblast growth factor receptor 3
H&E	Hematoxylin-Eosin
ICC	Immunocytochemistry
ITT	Immature testicular tissue
MAGEA4	Melanoma-associated antigen 4
PBS	Phosphate-buffered saline
PBST	PBS-Triton
SOX9	SRY-Box 9
TBS	Tris-buffered saline
TBST	TBS/Tween
