# Supplementary material for: Optimized Recovery of Immature Germ Cells after Prepubertal Testicular Tissue Digestion and Multi-Step Differential Plating: A Step towards Fertility Restoration with Cancer-Cell-Contaminated Tissue
Source: Int J Mol Sci. 2023 Dec 30;25(1):521. doi: 10.3390/ijms25010521 (PMC10779385; doi:10.3390/ijms25010521)
Supplement: Supplementary file 1 [file ijms-25-00521-s001.zip › ijms-2709154-supplementary.pdf]

Table S1. Measurements of weight and volume of obtained neonatal porcine testes. n=12.

Testis	Litter	Age (days)	Weight (mg)	Volume (μL)	Weight/volume ratio
1	3	3	791.8	650	1.22
2	3	3	534.7	450	1.19
3	3	3	479.2	400	1.20
4	3	3	713.0	700	1.02
5	5	3	684.2	900	0.76
6	1	3	446.2	400	1.12
7	1	3	713.1	800	0.89
8	1	3	471.0	500	0.94
9	2	3	539.5	600	0.90
10	2	5	532.8	600	0.89
11	4	5	739.6	850	0.87
12	4	5	691.3	700	0.99
	Mean ± SD		611.37 ± 121.74	629.17 ± 169.84	1.00 ± 0.15
